# Ultrasound Assessment in Polycystic Ovary Syndrome Diagnosis: From Origins to Future Perspectives—A Comprehensive Review

**DOI:** 10.3390/biomedicines13020453

**Published:** 2025-02-12

**Authors:** Stefano Di Michele, Anna Maria Fulghesu, Elena Pittui, Martina Cordella, Gilda Sicilia, Giuseppina Mandurino, Maurizio Nicola D’Alterio, Salvatore Giovanni Vitale, Stefano Angioni

**Affiliations:** Division of Gynecology and Obstetrics, Department of Surgical Sciences, University of Cagliari, SS554, 4, Monserrato, 09042 Cagliari, Italy; gineca.amfulghesu@tiscali.it (A.M.F.); elena.pittui@gmail.com (E.P.); martina.cordella92@gmail.com (M.C.); gilda.sicilia@gmail.com (G.S.); mandagiusi@gmail.com (G.M.); maurizion.dalterio@unica.it (M.N.D.); salvatoreg.vitale@unica.it (S.G.V.); sangioni@yahoo.it (S.A.)

**Keywords:** PCOS, PCOM, 3D ultrasound, ovarian stroma, machine learning, sensitivity, specificity, artificial intelligence

## Abstract

**Background**: Polycystic ovary syndrome (PCOS) is the most prevalent endocrinopathy in women of reproductive age, characterized by a broad spectrum of clinical, metabolic, and ultrasound findings. Over time, ultrasound has evolved into a cornerstone for diagnosing polycystic ovarian morphology (PCOM), thanks to advances in probe technology, 3D imaging, and novel stromal markers. The recent incorporation of artificial intelligence (AI) further enhances diagnostic precision by reducing operator-related variability. **Methods**: We conducted a narrative review of English-language articles in PubMed and Embase using the keywords “PCOS”, “polycystic ovary syndrome”, “ultrasound”, “3D ultrasound”, and “ovarian stroma”. Studies on diagnostic criteria, imaging modalities, stromal assessment, and machine-learning algorithms were prioritized. Additional references were identified via citation screening. **Results**: Conventional 2D ultrasound remains essential in clinical practice, with follicle number per ovary (FNPO) and ovarian volume (OV) functioning as primary diagnostic criteria. However, sensitivity and specificity values vary significantly depending on probe frequency, cut-off thresholds (≥12, ≥20, or ≥25 follicles), and patient characteristics (e.g., adolescence, obesity). Three-dimensional (3D) ultrasound and Doppler techniques refine PCOS diagnosis by enabling automated follicle measurements, stromal/ovarian area ratio assessments, and evaluation of vascular indices correlating strongly with hyperandrogenism. Meanwhile, AI-driven ultrasound analysis has emerged as a promising tool for minimizing observer bias and validating advanced metrics (e.g., SA/OA ratio) that may overcome traditional limitations of stroma-based criteria. **Conclusions**: The continual evolution of ultrasound, encompassing higher probe frequencies, 3D enhancements, and now AI-assisted algorithms, has expanded our ability to characterize PCOM accurately. Nevertheless, challenges such as operator dependency and inter-observer variability persist despite standardized protocols; the integration of AI holds promise in further enhancing diagnostic accuracy. Future directions should focus on robust AI training datasets, multicenter validation, and age-/BMI-specific cut-offs to optimize the balance between sensitivity and specificity, ultimately facilitating earlier and more precise PCOS diagnoses.

## 1. Introduction

Polycystic ovary syndrome (PCOS) represents the most common endocrinopathy affecting women of reproductive age. This syndrome encompasses a wide range of clinical manifestations, such as acne, hirsutism, alopecia, menstrual irregularities, and ultrasound features indicative of polycystic ovarian morphology (PCOM) [1]. Overweight women are more likely to have PCOS, and often need dietary supplements and complementary strategies, especially during pregnancy [2], as hyperandrogenism and insulin resistance are key factors in developing metabolic disorders in PCOS [3]. Indeed, it is frequently associated with central adiposity, insulin resistance, obesity, metabolic disturbances, impaired folliculogenesis, and oxidative stress, all contributing to an increased cardiovascular risk profile. The diagnosis of PCOS requires the careful application of well-established diagnostic criteria, including clinical or biochemical hyperandrogenism, assessment of ovarian morphology, and evaluation of menstrual irregularities [4,5]. Its prevalence varies according to the diagnostic criteria considered. According to the Rotterdam criteria, the prevalence ranges between 4% and 21%, whereas using the National Institute of Child Health and Human Disease (NICHD) criteria, it is 2–3 times lower, reaching 4–6.6% [6]. Indeed, the Rotterdam criteria introduce two other phenotypes: patients with anovulation and polycystic ovarian morphology (PCOM) without signs of hyperandrogenism and patients with PCOM and hyperandrogenism but regular ovulatory cycles [7]. The first description of polycystic ovary syndrome dates back to the work of Stein and Leventhal, who described the association between infertility, amenorrhea, hirsutism, and obesity in patients with enlarged ovaries characterized by a “pearly” appearance [8]. Subsequently, various scientific societies presented different definitions. In 1992, the NIH/NICHD proposed the combination of clinical or biochemical hyperandrogenism and menstrual cycle abnormalities as diagnostic criteria for PCOS, explicitly excluding hyperandrogenism related to other pathologies. Given the absence of ultrasound assessment in that definition, in 2003, the Rotterdam Consensus group redefined the diagnosis of PCOS by requiring the presence of at least two of the following criteria: oligo-anovulation, clinical or biochemical hyperandrogenism, and polycystic echo-structure of the ovary [7]. The Androgen Excess Society (AES) of 2006 stated that clinical and biochemical hyperandrogenism had to be associated with ovarian dysfunction or polycystic ovarian morphology to diagnose PCOS [9]. PCOS is, therefore, marked by a broad heterogeneity of signs and symptoms, which has led to ongoing debate regarding its precise definition among experts in gynecological endocrinology and pelvic ultrasound. This diagnostic uncertainty, compounded by frequent inaccuracies in current methods, often makes the prospect of undergoing an ovarian ultrasound during adolescence particularly daunting [10,11]. Such apprehension is understandable, given that the negative impact of PCOS on quality of life is closely linked to risks of infertility, hyperandrogenism (manifesting as obesity, hirsutism, and acne), and depressive disorders. Additionally, teenagers may fear a diagnosis of other conditions like endometriosis, which is now increasingly detected at an earlier stage and which can further exacerbate infertility issues [12] or lead to various multi-organ complications [13]. An accurate diagnosis of PCOS is therefore crucial, especially in light of its association with an increased risk of cardiovascular diseases, endometrial hyperplasia, type 2 diabetes, endometrial cancer, and depression [14,15]. In addition to mood disorders such as depression, PCOS patients frequently exhibit cognitive alterations and other neuropsychological challenges, underscoring the syndrome’s multifaceted impact on mental health [16]. Recent advances in artificial intelligence (AI) have begun to transform diagnostic strategies in gynecology. It has recently been demonstrated that machine learning algorithms can significantly enhance the speed and accuracy of PCOS diagnosis by integrating ultrasound imaging with anthropometric and biochemical data [17]. This review provides a comprehensive overview of the historical development and advancements in ultrasound imaging for diagnosing PCOS, focusing on emerging technologies and their various applications.

## 2. Search Strategy

This article is a narrative review of the relevant literature on ultrasound-based diagnosis of PCOS. We searched PubMed and Embase for English-language articles published, focusing on the new ones using keywords such as ‘PCOS’, ‘Polycystic Ovary Syndrome’, ‘Ultrasound’, ‘3D Ultrasound’, ‘Ovarian Stroma’, ‘Artificial Intelligence’, and ‘Machine Learning’. We prioritized studies discussing diagnostic criteria, imaging modalities, and stromal assessment. Reference lists of key papers were also screened for additional relevant articles.

## 3. Ultrasound Features of the Polycystic Ovary

### 3.1. Early Definitions and Transabdominal Assessments

From its discovery to today, PCOM has undergone numerous definitional changes. Initially, ovarian morphology was evaluated exclusively through the laparotomic approach. The evolution of ultrasound techniques has facilitated a progressive modification of the diagnostic criteria for PCOM, making sonographic evaluation one of the pillars in diagnosing the syndrome [18]. The first ultrasound evaluations, which began in the 1980s, aimed to investigate a possible correlation between ovarian morphology and endocrinological alterations in women with this syndrome [19]. In 1985, Adams established that polycystic morphology could be defined by the presence, on transabdominal ultrasound, of at least 10 small follicles (average diameter 2–9 mm) arranged subcortically in a single section, along with increased stromal density and ovarian volume (≥8 mL) [20].

### 3.2. Rotterdam Criteria and Transvaginal Ultrasound

In 2003, the Rotterdam Consensus [7] supported by the advent of the transvaginal ultrasound (TV-US) approach, and a careful review of the available literature defined the sonographic diagnosis of PCOM by the presence of at least one of the following criteria:

- ≥12 or more follicles in at least one ovary, considering all the follicles present from the inner to the outer edge in different sections.
- Follicular diameter between 2 and 9 mm (measured as the average of three diameters in longitudinal and transverse planes or considering the diameter of a circular-appearing follicle in the scan).
- Increased OV in at least one ovary (>10 cm^3^).

The calculation of ovarian volume involves identifying three diameters and using the ellipsoid formula π/6 × (length × width × thickness) or the simplified ellipsoid formula 0.5 × (length × width × thickness). Experienced operators should perform ultrasound during the early follicular phase or, in the case of oligo/amenorrheic patients, at a random period or 3–5 days after the onset of bleeding induced by progesterone. Detecting a dominant follicle (>10 mm), pathological cysts, or hormonal therapy in progress precludes a correct evaluation of ovarian morphology, thus requiring a re-evaluation in the subsequent early follicular phase [7]. A TV-US is generally preferred over a transabdominal ultrasound (TA-US) in assessing ovarian morphology due to its higher resolution, closer proximity to the ovaries, and superior ability to delineate follicular structures. This modality provides a more detailed and reliable visualization, particularly critical in evaluating PCOS [21].

### 3.3. Anatomical Overview of the Ovaries

The ovary is a paired reproductive organ with a complex internal structure. It consists of an outer cortex, where follicles in various stages of development are located, and an inner medulla that contains blood vessels and connective tissue. The ovarian cortex is the site of follicular maturation, with each follicle comprising an oocyte surrounded by granulosa and theca cells. This anatomical organization is crucial for normal ovarian function and understanding the morphological changes in PCOS, where increased follicle count and altered stromal density are often observed [22].

### 3.4. Follicle Number and Ovarian Volume Thresholds

The ovarian volume (OV) can be calculated via the ellipsoid formula on standard ultrasound systems or with three-dimensional approaches. Unlike follicle numbers per section (FNPS) and follicle number per ovary (FNPO), the OV cut-off has changed little since the 2003 Rotterdam definition, which set a threshold of >10 cm^3^ [23]. Nevertheless, several groups have proposed potentially lower cut-offs (ranging from 6.4 to 7.5 cm^3^) modulated by ethnicity, body mass index, and insulin levels [24]. Clinicians must also consider the patient’s age, as the ovary grows during childhood, peaks shortly after puberty, and starts decreasing significantly after around 30 years of age [25]. A study by Fruzzetti et al. showed distinct adolescent-specific thresholds for enlarged ovaries, 11.5 cm^3^ within the first 2 years post-menarche, 10.5 cm^3^ in the third year, and 10 cm^3^ in the fourth/fifth years, converging with adult cut-offs after about 2 years of menstrual function [26]. Diagnosis of PCOM relies chiefly on OV, FNPS, and FNPO [27]. At least 12 antral follicles (2–9 mm) in one ovary remains one of the principal criteria of the Rotterdam consensus and allows a good distinction between controls (mean OV 5.8 and 6.4 mL) from PCOM patients (mean OV 10.6 to 16.7) [28]. However, progressive improvements in imaging have led to revised follicle-number thresholds, since 30–50% of normal-androgenic, ovulatory women may already surpass the older ≥12-follicle threshold when using high-frequency probes) [27,29]. Indeed, Lujan et al. proposed 26 follicles (on a transvaginal transducer ≥8 MHz) as a more accurate cutoff to distinguish PCOM from normal ovaries [27]. In 2014, the AES-PCOS Society recommended ≥25 follicles per ovary for high-frequency probes [24]. Likewise, the 2018 and 2023 international guidelines recommend ≥20 follicles in at least one ovary as pathological [30,31]. Although these more significant cut-offs might reduce overdiagnosis, they are difficult to implement widely because many clinical settings lack high-frequency transvaginal probes or must rely on transabdominal scans, particularly in adolescents [27]. Moreover, a recent meta-analysis from the Italian Society of Gynaecology and Obstetrics and the Italian Association of Endocrinology indicated that the original Rotterdam thresholds (≥12 follicles or OV > 10 cm^3^) remain substantially reliable, as healthy women still tend to stay below these numbers—even with higher-resolution technology [32]. Follicle distribution, stromal echogenicity, and stromal area can be helpful adjuncts in clinical practice, but are not included among the core diagnostic criteria. These features can, nonetheless, be relevant in refining a patient’s risk profile and guiding individualized follow-up. Multiple tools, such as segmenting the ovary into quadrants or using three-dimensional ultrasound (3D-US), have also been investigated to reduce operator-dependent variability in follicle counts [33]. Table 1 summarizes progress in ultrasound-based diagnostic criteria for polycystic ovarian morphology.

### 3.5. Sensitivity and Specificity of Ultrasound in PCOS Diagnosis

One of the earliest and most frequently cited ultrasound definitions of polycystic ovaries was introduced by Adams et al. (1985), who used ≥10 follicles (2–8 mm) in a single cross-section of the ovary on transabdominal ultrasound. However, robust sensitivity and specificity for this criterion were not clearly reported [34]. In 2003, Jonard et al. proposed ≥12 follicles (2–9 mm) by applying receiver operating characteristic (ROC) curves, attaining about 75% sensitivity and 99% specificity in distinguishing PCOS from non-PCOS ovaries, helping to shape the Rotterdam criteria in the same year [28]. Subsequent work revealed that when high-frequency (≥8 MHz) transducers are employed, up to 30–50% of otherwise healthy, normo-androgenic women might exceed the older 12-follicle threshold [29]. Consequently, the Androgen Excess and PCOS (AEPCOS) Society suggested ≥25 follicles (while retaining OV ≥ 10 cm^3^) [24]. The latest Evidence-Based Guidelines then put forward ≥20 follicles as a compromise, underscoring that definitive sensitivity/specificity values for either 25 or 20 follicles remain under continuous investigation [30,31]. In addition to follicle counts, ovarian volume (OV) has also demonstrated diagnostic utility. Specifically, an OV > 10 cm^3^ has been associated with an approximate 81% sensitivity and 84% specificity when evaluating PCOM, though it may be less specific than counting follicles in well-visualized ovaries [27]. Conversely, an FNPO threshold of ≥12 provides a better trade-off, with ~75% sensitivity and 99% specificity [28]. Lujan et al. proposed 26 follicles as an alternative cutoff, reporting 85% sensitivity and 94% specificity, although real-world applications can be challenging [27]. Overall, increasing the follicle-number threshold generally improves specificity (i.e., fewer false positives) at the expense of reduced sensitivity, since some women with genuine PCOS may have borderline follicle counts. Moreover, factors such as age, ethnicity, BMI, and probe frequency can affect the reliability of each cutoff. Many clinicians, therefore, emphasize that hyperandrogenism and ovulatory dysfunction often correlate more strongly with metabolic outcomes than ultrasound findings alone [35,36]. As imaging methods continue to improve, and large-scale, standardized investigations are conducted, it is anticipated that the diagnostic performance of ultrasound criteria will be further refined, ensuring that PCOM remains a robust and reliable part of the PCOS diagnostic landscape.

### 3.6. Follicle Distribution and Size

Regarding follicle distribution, PCOM was historically described as two distinct ultrasound patterns: the peripheral cystic pattern (PCP) and the general cystic pattern (GCP). In PCP, follicles are arranged in the subcortical region in a “string of pearls”, whereas in GCP, they are distributed diffusely throughout the ovarian parenchyma. PCP has shown a stronger association with menstrual irregularity. Studies indicate that 94% of subjects with bilateral PCP experience oligomenorrhea, compared to 65% with unilateral PCP. About 29% of women with bilateral GCP also present menstrual disturbances, but the prevalence is lower than bilateral PCP and similar to unilateral PCP. Serum testosterone levels and LH/FSH ratio are significantly higher in patients with PCP distribution than in those with GCP [37]. Despite these observations, follicle distribution has not been fully incorporated into official guidelines. Interestingly, it has also been hypothesized that women with PCOS display an intrinsic alteration in folliculogenesis, as they exhibit an excess of follicles measuring 2–5 mm rather than 6–9 mm, demonstrating an excessive initial follicular growth associated with an early arrest [28]. These smaller follicles (2–5 mm) correlate positively with serum testosterone and androstenedione levels [38]. Although PCOM on ultrasound is part of the Rotterdam criteria for adult women, its use in adolescence is not recommended to avoid overdiagnosis [31]. Indeed, 40% of patients at 2 years post-menarche, 35% at 3 years, and 33.3% at 4 years display PCOM using the Rotterdam cut-off. Hence, PCOM may represent a transient phase of ovarian development in early adolescence, potentially reflecting an immature hypothalamic–pituitary axis or functional hypothalamic amenorrhea [39].

## 4. The Role of Ovarian Stroma

**Figure 1:**
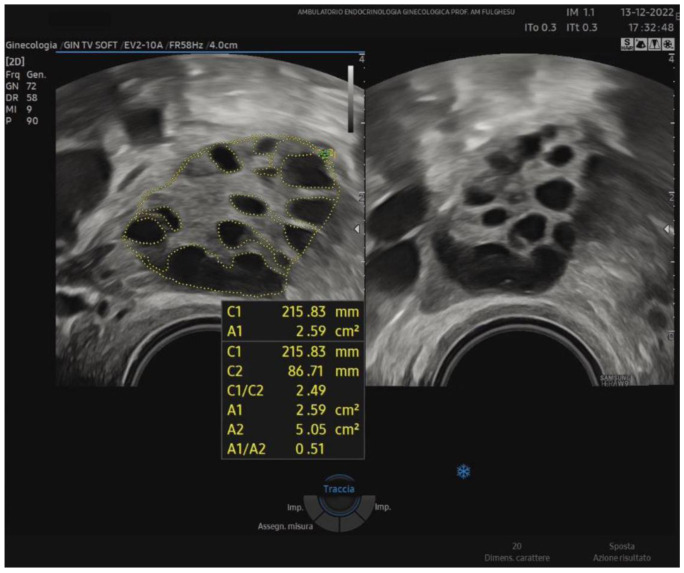
Example of the SA/OA ratio calculation on a median ovarian section with outlined ovarian and stroma areas during a TV-US scan. A1: total stromal area; A2: ovarian area; A1/A2: SA/OA ratio (>0.32).

Recent investigations focus on new ultrasound markers to better characterize ovarian morphology. One method is the stromal area/ovarian area (SA/OA) ratio, which, with a cut-off of 0.32, has been proposed as a significant predictor of elevated androstenedione and testosterone levels [40]. Because the Rotterdam criteria are not recommended for adolescents due to transabdominal scans and the high prevalence of PCOM in healthy teens, some authors have suggested measuring the SA/OA ratio to reduce the risk of overdiagnosis. In studies involving normocyclic and non-hyperandrogenic adolescent girls, applying the Rotterdam guidelines (follicle count ≥ 12, OV > 10 mL) and setting the standard SA/OA ratio to ≤0.3 led to the classification of ovaries into three groups: normal morphology (NOM), PCOM with normal SA/OA (PCOM-NS), and PCOM with increased SA/OA (PCOS-IS). Notably, the prevalence of PCOM-IS remained constant across different post-menarche ages, indicating that an increased stromal ratio might be a more stable feature associated with androgen excess. Indeed, PCOM may be considered a normal and transient physiological phase in ovarian development during early adolescence, predominantly observed in the first 1–3 years post-menarche because of an immature hypothalamic-pituitary axis [41,42]. Around 1985, Adams et al. noted the characteristic peripheral arrangement of follicles around a core of hyperechoic stroma [34]. Similarly, Dewailly in 1994 observed that ovarian hypertrophy, indirectly linked to stromal hypertrophy, was easy to measure and correlated with PCOS [43]. In recent decades, numerous attempts have been made to quantify stromal echogenicity and thickness more objectively. For instance, stromal and myometrial echogenicity have been compared, considering that normal stromal echogenicity should be slightly lower than the myometrium. However, operator subjectivity and equipment differences have hindered standardization. In 2001, Fulghesu et al. [44] proposed quantifying the stromal percentage in the central zone using calipers to outline the ovarian periphery and the stromal core on a still ultrasound image corresponding to the maximum planar section of the ovary (SA/OA ratio) (Figure 1).

With this type of measurement, values of SA/OA > 0.32, which reflect a stroma occupying more than one-third of the ovary in the midsection, suggest a more pronounced androgenic profile, corresponding to a higher likelihood of PCOS. The stromal area-to-total ovary area ratio can be assessed without the need for other technologies using standard ultrasound and eliminates the problems of subjective stromal assessment encountered previously. Belosi et al. [45] confirmed that the SA/OA ratio shows minimal inter-operator variability, high diagnostic accuracy, and a strong correlation with plasma androgen levels. Moreover, by adding SA/OA ratio measurement, one can identify subgroups of patients with borderline or NIH-negative status who nonetheless display underlying hyperandrogenism. In 2007, a multicenter study in Italy corroborated these findings, demonstrating that the SA/OA ratio best predicts elevated androstenedione and testosterone levels among the various ultrasound parameters [40]. In conclusion, ultrasound-based diagnosis of PCOS still relies on the Rotterdam criteria (2003). However, adding a stromal evaluation (SA/OA ratio) may significantly improve the identification of hyperandrogenic phenotypes. Quantifying both stromal area and follicle distribution is crucial to accurately diagnosing PCOS, offering the possibility of distinguishing different phenotypes and optimizing patient management.

## 5. Three-Dimensional (3D) Transvaginal and Transabdominal Ultrasound

**Figure 2:**
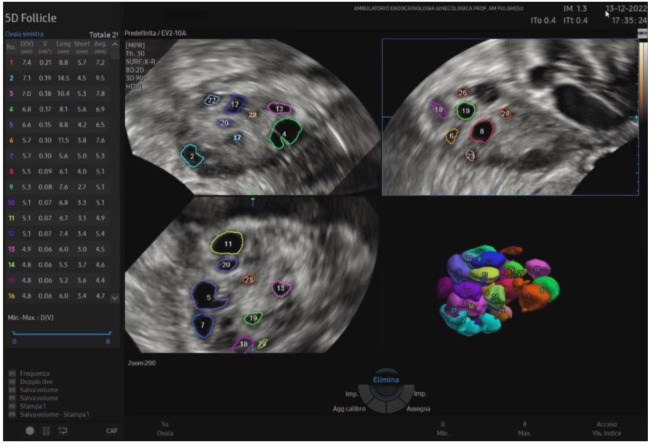
A 3D study of the ovary: identification and measurements of the ovarian follicles to rapidly assess the size, follicular status, and spatial arrangement.

Recently, 3D ultrasound has gained increasing popularity in studying PCOS and reproductive medicine. It appears to provide a more reliable and objective assessment of ovarian volume and morphology, stromal area, follicle count, and overall blood flow than traditional 2D methods, as shown in Figure 2.

**Figure 3:**
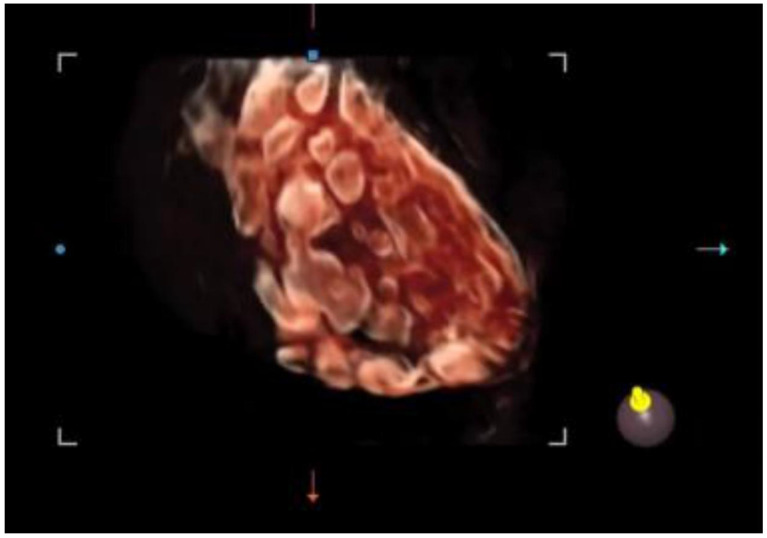
Three-dimensional ultrasound scan using the inversion mode.

It offers a noninvasive, safe, and painless option for evaluating the internal genitalia in young women. Moreover, 3D ultrasounds can detect more follicles than 2D imaging, enhancing diagnostic accuracy [46,47]. In adolescents, where the diagnosis of PCOS remains uncertain after clinical and laboratory evaluation, magnetic resonance imaging (MRI) could be considered an alternative imaging modality. However, if 3D-TA approaches become widely available, with suitably high-resolution probes, 3D ultrasound may reduce the reliance on MRI in borderline cases [48]. Among the recently employed three-dimensional ultrasound techniques in evaluating the PCOM, inversion mode provides a highly effective way to highlight fluid-filled structures such as antral follicles. By virtually inverting the echogenic signals, the typically hypoechoic areas (e.g., follicular fluid) appear hyperechoic, allowing for easier follicle detection and quantification (Figure 3). This method can reduce the error in follicle counting, enhance the visualization of the follicular layout, and provide additional insights into the stromal–follicular relationship [49].

### 5.1. Advantages of 3D Software and Automated Follicle Measurement

The automatic measurement of follicular diameter using dedicated 3D software (HS40 s/w v.1.02) has multiple advantages over the traditional 2D technique. First, it shortens examination time since ultrasound data can be stored in toto and analyzed later. These data can be reconstructed in any plane, independent of the original scanning orientation, allowing for a more detailed and flexible evaluation. Furthermore, this innovative method reduces operator dependence in interpreting images, enhancing objectivity and minimizing interobserver variability [50]. A recent study in overweight PCOS patients supports this evidence. It showed that 2D TV-US underestimated ovarian volume and antral follicle count (AFC) compared to 3D TV-US and MRI [31]. Therefore, 3D ultrasound appears more accurate than 2D methods in specific populations, including overweight women, and may improve the diagnostic sensitivity for PCOS [51].

### 5.2. Ultrasound vs. MRI

Pelvic MRI offers additional diagnostic advantages by providing high-contrast resolution and multiplanar imaging capabilities. In T1-weighted sequences, MRI can effectively identify fatty tissue and hemorrhagic areas, while T2-weighted sequences are particularly adept at visualizing fluid-filled structures such as ovarian follicles. The distinct signal characteristics on T1 and T2 images can help differentiate normal ovarian tissue from pathological changes associated with PCOS. Although ultrasound remains the first-line imaging modality due to its accessibility and cost-effectiveness, MRI can be a valuable complementary tool in cases where ultrasound findings are inconclusive [52]. Although some studies have compared 3D ultrasonography and MRI scans of the ovaries, only a limited number include TA (rather than TV) US. Hagen et al. considered follicles measuring down to 1 mm in the category of small follicles, and found a comparable number of follicles > 4 mm using MRI and 3D US [53]. This suggests that 3D US can rival MRI in accuracy for specific follicle size ranges.

### 5.3. Stromal Evaluation and Vascularization with 3D Ultrasound

Studies using new 3D ultrasound technology indicate that the ovarian stromal area and the stromal/ovarian area (S/A) ratio are significantly higher in PCOS patients compared to controls, and these parameters correlate with androgen levels and hirsutism. The thickening of the ovarian stroma likely reflects the prominent theca and fibrotic thickening of the luteal cell albuginea. Since theca cells within the ovarian stroma are responsible for androgen production, stromal hypertrophy plays a key role in PCOS pathophysiology. Nevertheless, the universal acceptance of stromal hypertrophy as a diagnostic criterion remains controversial [33,44]. Despite this controversy, the data strongly support using 3D ultrasound to evaluate the SA/OA ratio in diagnosing PCOS, as it seems to be the parameter most strongly correlated with androgen levels. Indeed, building on this perspective, Kinnear et al. highlight that ovarian stroma is an emerging research frontier, with diverse cell populations and extracellular matrix components critically influencing ovarian physiology and potentially driving PCOS pathology [54].

### 5.4. Three-Dimensional Ultrasound in Adolescents and Obese Patients

In adolescents who are not sexually active, pelvic ultrasonography is usually performed transabdominally. Sujata et al. found that 2D and 3D scans are equally accurate in assessing ovarian morphology [55]. Still, the resolution may be suboptimal in overweight/obese individuals due to the increased distance between the probe and the ovary, which complicates the evaluation of ovarian morphology. Moreover, the use of 2D US to assess stromal echogenicity has faced criticism for subjectivity and limited reproducibility. By contrast, 3D US permits simultaneous visualization of the ovary in three orthogonal planes, enabling direct measurement of ovarian stromal volume (i.e., total ovarian volume minus total follicular volume) and being generally less subjective than 2D US and allows a more objective, precise evaluation of OV, SA, OA, and overall blood flow [33]. The hyperechogenicity of the ovarian stroma in patients with PCOS helps differentiate multicystic ovaries, which are generally seen in adolescents [56]. However, further research is essential to establish whether 3D-TA scans can reliably measure the SA/OA ratio in obese and non-obese adolescents. Table 2 shows a brief comparison between the 2D US and 3D US.

### 5.5. Three-Dimensional Power Doppler and Ovarian Vascularization

Studies have demonstrated increased ovarian blood flow in PCOS patients [57,58], with 3D power Doppler indices significantly higher than in controls [33]. Furthermore, total ovarian vascularization index, flow index, and vascularization flow are considerably higher in normal-weight PCOS women than their overweight counterparts [56,59,60]. Vascularization analysis might also be a valuable tool in evaluating infertility and the response to fertility treatments [61,62]. The elevated luteinizing hormone may partly explain this increased stromal vascularization through various mechanisms, including neoangiogenesis, catecholaminergic stimulation, leukocyte, cytokine activation, and high vascular endothelial growth factor levels [33,56]. Consequently, 3D Doppler imaging that measures these vascular indices could be another key marker to improve PCOS diagnosis. Indeed, in their study of women meeting the Rotterdam criteria for PCOS, Lam et al. (2007) demonstrated that 3D US quantification of ovarian stromal volume and vascularity (VI, VFI) distinguishes polycystic from normal ovaries and highlights phenotypic variations, with higher stromal vascularity observed in hirsute or normal-weight women [63]. In a recent systematic review and meta-analysis, Wang et al. highlighted that an advanced 3D Doppler ultrasound reveals significantly higher ovarian vascularization and flow indices (VI, FI, VFI) and lower resistance indices (PI, RI) in women with PCOS compared to controls, underscoring its utility in detecting altered ovarian blood flow [64].

### 5.6. Integrating 3D Ultrasound in PCOS Diagnosis: A Practical Flowchart

**Figure 4:**
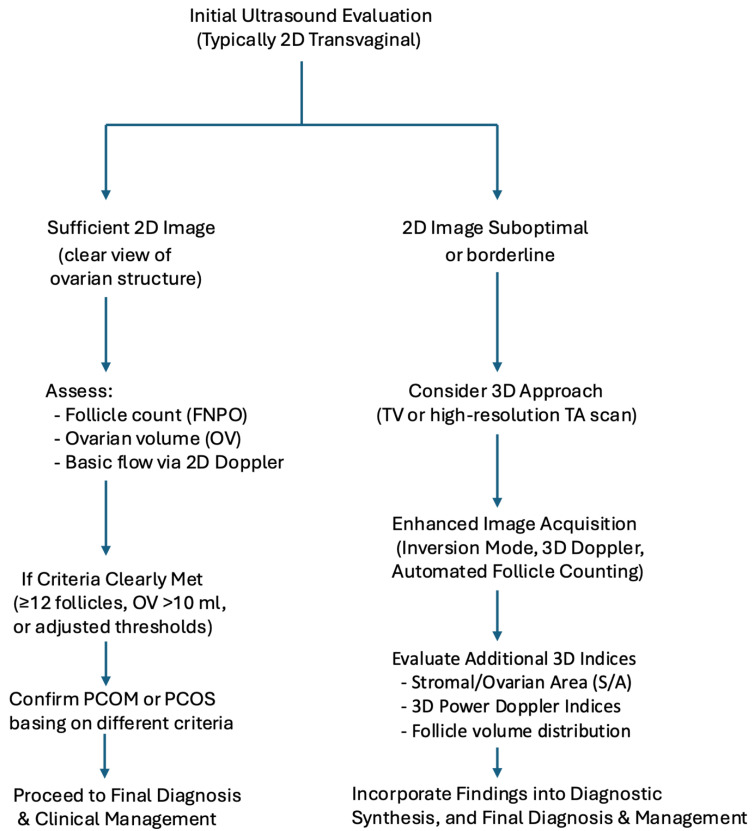
Flowchart of diagnostic and ultrasound evaluation approaches in PCOS, combining 2D and 3D US.

Figure 4 illustrates how 3D US can be selectively employed in the diagnostic pathway for PCOS rather than relying solely on conventional 2D scans. When the standard 2D TV US, including FNPO and OV measurements, yields precise results, further imaging may not be necessary. However, in several situations, 3D US can offer significant advantages:

1. Suboptimal 2D imaging: adolescents requiring TA scans due to lack of sexual activity, obese patients with poor acoustic windows, or borderline follicle counts that raise doubts about the final PCOS classification.
2. Advanced quantifications: the 3D mode enables automated follicle segmentation, improved smaller or overlapping follicles detection, and a more precise inversion mode to highlight fluid-filled structures.
3. Stromal assessment: the SA/OA ratio and 3D power Doppler indices (vascularization index, flow index, etc.) can be measured more consistently, particularly in overweight/obese patients or borderline cases.

If the 2D US clearly demonstrates ≥12 follicles and/or an OV > 10 cm^3^ (or the revised thresholds for high-frequency probes), one might finalize the morphological diagnosis without 3D.

If 2D imaging is borderline (e.g., 10–11 follicles per ovary) or technically challenging (low resolution, uncertain follicle size), the 3D approach offers additional clarity, potentially preventing misclassification and enabling more accurate phenotyping.

This approach maximizes efficiency and diagnostic accuracy in PCOS assessment by guiding clinicians to “switch” to 3D only when 2D results are inadequate or ambiguous.

## 6. Perspectives on AI-Driven Ultrasound Analysis in PCOS

AI and machine learning have gained increasing attention as ultrasound technology evolves, due to their potential to develop predictive analytics tools that help standardize and automate diagnostic imaging evaluation, including PCOM [65]. New integrated pre-processing techniques and novel feature extraction via innovative algorithms may be used to detect and classify PCOS from ultrasound images with high diagnostic accuracy, thereby streamlining the diagnostic process and reducing the reliance on manual interpretation [66]. Similar approaches integrating artificial intelligence and machine learning have already been successfully applied in other radiology fields, such as diabetic retinopathy screening and breast imaging, where these technologies have significantly enhanced diagnostic accuracy and efficiency [67,68]. In principle, validated ultrasound criteria, such as follicle number per ovary, ovarian volume cut-offs (e.g., >10 cm^3^), and emerging stromal indices, could serve as training targets for AI algorithms. By exposing these models to large, annotated databases of ultrasound images (encompassing both PCOM and non-PCOM examples), the AI can learn to identify and quantify key morphological features [69]. The process would begin with feature extraction, in which automated segmentation tools detect and measure ovarian follicles, ovarian volume, and potentially stromal echogenicity or vascular indices. Once the machine learning system has been trained on large, heterogeneous, and multicentric datasets, it could classify new, unseen ultrasound scans with reduced operator dependence, improving consistency and maintaining or increasing sensitivity and specificity relative to human interpretation. Although the Rotterdam criteria combined with transvaginal ultrasound demonstrate high sensitivity (87–95%), these methods remain subject to operator-dependent variability and subjective interpretation, particularly in borderline cases. AI integration offers the potential for objective, reproducible, and quantitative assessment, which may further standardize diagnostic evaluations.

For instance, Suha et al. developed an extended machine-learning technique for PCOM detection, reaching a maximum accuracy of 99.89% with a relatively short execution time to detect PCOS from ultrasound images [70]. Such an approach addresses one of the central challenges in PCOS diagnostics: operator-related inter- and intra-observer variability. Current guidelines and cut-offs (e.g., ≥12 vs. ≥20 follicles, 10 cm^3^ vs. 7.5 cm^3^ ovarian volume) might be incorporated into multimodal AI platforms, allowing them to fine-tune classification thresholds based on imaging parameters, patient age, and BMI. The next logical step is to extend these methods to quantify stromal parameters, particularly the SA/OA ratio. Historically, measuring the stromal region by manual tracing has shown promise for discriminating hyperandrogenic states in PCOS [44,45]. Yet, this approach has never been universally adopted due to significant operator dependence and lack of complete reproducibility in results [71]. Indeed, although a SA/OA ratio > 0.32 has been confirmed to correlate with biochemical hyperandrogenism and implemented with the 3D-US analysis [33], variability in the acquisition planes and the operator’s subjective selection of stromal boundaries has prevented the widespread acceptance of stroma-based parameters as additional diagnostic criteria.

By leveraging AI-driven segmentation of the ovary and its internal structures, an automated platform could consistently define the ovarian boundary and the reflective stromal region, providing an objective SA/OA ratio for each image or 3D dataset. Such an approach would effectively bypass human inter-operator discrepancies, unify existing validated thresholds (e.g., >0.32), and offer new cut-offs derived from larger, multi-center datasets. Once trained on sufficiently varied ultrasound images captured by different devices and operators, this model could detect subtle textural and echogenic differences that delineate stroma from surrounding follicular tissue, further refining PCOS phenotyping. If validated in robust prospective studies that demonstrate improved diagnostic accuracy over older methods, an automated stromal ratio could reestablish stroma as an essential diagnostic factor and highlight it as an exciting new frontier for research [54]. Ultimately, combining traditional criteria (follicle count, ovarian volume) with an AI-based stroma metric could help overcome the historical barriers that prevented stroma assessment from becoming standard in PCOS diagnostics, leading to a more comprehensive and objective classification of ovarian morphology in PCOS. Specific foundational steps are necessary before AI-guided methods can be used routinely. These include creating large, standardized ultrasound datasets, thorough external validation for broad applicability, and transparent regulatory oversight on privacy and ethics. As these requirements are addressed, AI-driven ultrasounds may significantly reduce diagnostic ambiguity, enhancing traditional clinical and biochemical evaluations to yield a more comprehensive and objective PCOS diagnosis. AI-based systems are designed to serve as decision-support tools rather than autonomous diagnostic entities. The interpretation of AI-generated outputs must be reviewed and validated by experienced clinicians to ensure appropriate patient management.

## 7. Future Directions and Limitations

Ultimately, advanced ultrasound techniques, particularly 3D-US, offer new opportunities for objective and quantitative evaluation of ovarian morphology, stromal hypertrophy, and vascular parameters in PCOS, refining existing diagnostic criteria. As technology evolves, integrating 3D approaches into routine practice may promote earlier, more accurate PCOS diagnoses, enhanced phenotyping, and greater personalization in treatment strategies [72]. Despite the notable progress of 3D ultrasound, several constraints persist, including high equipment costs, a need for specialized training, and the technical challenge of achieving high-resolution images transabdominally, particularly in adolescents and obese patients. In this context, further research is crucial to validate the clinical feasibility, reliability, and cost-effectiveness of 3D transabdominal ultrasound for adolescent ovaries, with special emphasis on the stromal area/ovarian area (SA/OA) ratio and vascularization. Furthermore, integrating AI-driven solutions could help standardize imaging protocols, reduce inter-operator variability, and ensure cutting-edge ultrasound techniques become more accessible and reproducible in everyday clinical practice. While these techniques may exclude scenarios in resource-poor settings due to the technical complexity and high cost of equipment, advancements in AI software may lessen the need for hardware, significantly decreasing the cost of this promising new technology. Nevertheless, future prospective studies, including well-designed clinical trials with sufficient sample sizes, are needed to validate the diagnostic benefit of AI integration over standard ultrasound techniques. The limitations of this review include the heterogeneity of the included studies concerning sample sizes, methodologies, and diagnostic criteria. Many of the reviewed studies rely on retrospective data, which may introduce bias and limit the broader application of the findings. Additionally, while emerging AI-based approaches in ultrasound imaging demonstrate promising results, most remain preliminary and need further prospective validation in larger, multicentric studies. These limitations underscore the necessity for standardized protocols and more rigorous research to fully establish the clinical utility of these advanced diagnostic tools in PCOS.

## Figures and Tables

**Table 1:** Ultrasound and PCOM evolution through the years.

1985-Adams J.	2003-Rotterdam Criteria	2014-Task Force Report from the AE-PCOS Society	2023-Evidence-Based Guideline for the Assessment and Management of PCOS
Transabdominal US	Transvaginal US	Transvaginal US (>8 mHz)	Transvaginal US (>8 mHz)
FNPS > 10 (2–8 mm) Follicles peripherally distributed Dense stroma	FNPS > 12 (2–9 mm) in at least one ovary OV > 10 mL	FNPO > 25 (2–9 mm) in at least one ovary	FNPO > 20 (2–9 mm) in at least one ovary
-	-	OV > 10 mL using older technology	FNPS > 10 or OV > 10 mL using older technology

**Table 2:** Two-dimensional US vs. Three-Dimensional US.

Parameter	2D Ultrasound	3D Ultrasound
Acquisition Time	Real-time scanning can be longer	Data volume can be acquired quickly; analysis can happen offline
Follicle Count Accuracy	Often underestimates AFC, especially in obese PCOS	Generally more accurate, can detect smaller follicles and minimize overlap errors
Interobserver Variability	Moderate to high	Lower, due to automated software and reconstruction
Utility in Obese Patients	Decreased resolution transabdominally	It would potentially better if high-frequency 3D probes are available
Equipment Cost	Relatively lower	Higher specialized probes and training required

## Data Availability

The original contributions presented in the study are included in the article, further inquiries can be directed to the corresponding author.
